# Late-onset volvulus without malrotation in extremely preterm infants - a case–control-study

**DOI:** 10.1186/s12887-014-0287-2

**Published:** 2014-11-12

**Authors:** Christoph Maas, Stefanie Hammer, Hans-Joachim Kirschner, Yasemin Yarkin, Christian F Poets, Axel R Franz

**Affiliations:** Department of Neonatology, University Children`s Hospital, Calwerstr. 7, 72076 Tuebingen, Germany; Department of Paediatric Surgery and Paediatric Urology, University Children`s Hospital, Tuebingen, Germany

**Keywords:** Intestinal volvulus, Acute abdomen, Newborn, Infant premature, Necrotising enterocolitis

## Abstract

**Background:**

Volvulus without malrotation in preterm infants is a rare but potentially life-threatening event of unknown aetiology. Confusion with necrotising enterocolitis may delay surgical intervention thereby aggravating morbidity and mortality.

We aimed at elucidating potential risk factors for, and characteristic clinical signs of, volvulus without malrotation in preterm infants.

**Methods:**

Retrospective, single-centre case–control study (2007–2011). For every index patient, five infants of similar gestational age, birth weight and birth year were evaluated. Additionally, all 9 cases of necrotising enterocolitis occurring during the above period were evaluated. Data are presented as median (interquartile range).

**Results:**

Five extremely premature infants suffering from volvulus without malrotation were identified (gestational age at birth 24.4 (23.6-25.5) weeks, birth weight 480 (370–530) g). All were small for gestational age and female; three out of five died. Volvulus occurred several weeks after birth, whereas necrotising enterocolitis occurred significantly earlier. Beyond that, no striking differences in clinical or laboratory presentation of volvulus without malrotation and necrotising enterocolitis were found. Infants with volvulus had significantly more frequent manipulations with rectal tubes for flatulence, but there were no differences in the frequency of enemas, abdominal massage or defecation. In infants with volvulus, nasal high-frequency oscillation was used more frequently for respiratory support, and PEEP-level tended to be higher.

**Conclusions:**

In extremely premature infants volvulus without malrotation represents a life-threatening event that occurs typically several weeks after birth with an acute abdomen and seems to affect predominantly girls. Infants requiring intensive non-invasive respiratory support might be at highest risk.

## Background

Intestinal volvulus in association with malrotation is a well recognized condition in the newborn [1] and an important cause of gastrointestinal emergency in the preterm infant [2].

In contrast, primary volvulus without malrotation (VWM) is expected to be a very rare event. However, there is no reliable data on the incidence of VWM. Case series on primary VWM in the perinatal period report a predominance of infants born prematurely [3-7], particularly before 30 weeks gestational age and with a birth weight <1000 g; many were affected several weeks after birth. Furthermore, there are case reports that describe the occurrence of VWM in utero [7-9].

VWM generally presents as an acute abdomen with a rapid and dramatic deterioration of the infant’s general condition. In some infants, repeated episodes of abdominal distension and/or subacute intestinal obstruction were described as early clinical signs preceding the manifestation of VWM [4]. Especially in the vulnerable population of very preterm infants, diagnosis before the occurrence of irreversible intestinal ischemia may be difficult due to a lack of specific radiological or ultrasound features revealing the underlying strangulating obstruction [4] and the rapid progression of ischemic bowel damage. Additionally, volvulus may be mistaken for the more common entity of necrotising enterocolitis (NEC) [2] which may delay the surgical intervention thereby aggravating morbidity and mortality. The ischemic changes of the twisted bowel in VWM are supposed to progress more rapidly because of a normal position and fixation of the uninvolved colon, whereas in cases of volvulus with malrotation and mobile cecum, the colon may attenuate ischemia and the sequelae of small bowel torsion [10-12].

Intrauterine foetal demise due to midgut volvulus is even more infrequent but may be of similar aetiology [13].

The aetiology of VWM is unknown [12]. The absence of a segment of small bowel musculature or a mesenteric defect have occasionally been described in VWM [14,15]. Furthermore, respiratory support with CPAP as well as abdominal massage and pelvic rotation were suspected as risk factors [3,4,6].

The aim of this case–control-study was to re-evaluate potential risk factors and characteristic early clinical signs of VWM in very preterm infants to potentially inform strategies for prevention.

## Patient and methods

This retrospective case–control study was performed at Tübingen University Children’s Hospital. The ethics committee at the University of Tübingen, Faculty of Medicine, approved the retrospective evaluation and waived the need for parental consent, hence parental consent was not asked for.

All five inborn preterm infants with VWM identified among 554 life born infants with a birth weight <1500 g (very low birth weight (VLBW) infants) at our institution between January 1, 2007, and December 31, 2011 were included. There have been no further cases of VMN until July 2014 (another 277 VLBW infants). For each case, five control infants without VWM or gastrointestinal conditions entailing surgical procedures were selected from inborn infants (n = 507), matched for gestational age at birth, birth weight and year of admission, a priori excluding infants who had undergone laparotomy. Additionally, all 9 inborn VLBW infants with NEC Bell stage ≥2 in this period (2007–2011) were evaluated.

Medical charts of included infants were meticulously analysed for pre-defined potential risk factors and clinical signs derived from previous reports and our own experience. Data included demographic variables, neonatal morbidities, parameters of gastrointestinal function, data on initiation, advancement and type of enteral nutrition, manipulations intended to promote regular gastrointestinal transport, duration, type and intensity of respiratory support, frequency and intensity of chart-documented apnoea of prematurity, clinical signs and laboratory abnormalities on the day of presentation as well as any administration of medications with suspected or proven gastrointestinal interactions. In control infants, these parameters were evaluated at the equivalent postnatal age of VWM-presentation in the corresponding index infant as well as pre-defined time periods prior to the presentation (prodromal stage). These pre-defined time periods included ten to four days (d-10 – d-4) and three to one days (d-3-d-1) prior to presentation.

### Statistical analyses

Data are presented as medians (interquartile range). To evaluate weight gain, standard deviation score (SDS) for weight was computed using the Microsoft Excel add-in LMS Growth (version 2.14; http://www.healthforallchildren.com/?product=lmsgrowth). The reference population for this program is the British 1990 growth reference fitted by maximum penalized likelihood [16,17]. Comparisons between cohorts were performed using the Wilcoxon/Kruskal-Wallis test or fisher`s exact test. Statistical significance was assumed at p <0.05. Analyses were performed with JMP® 11.1.1 (SAS Institute Inc., USA).

## Results

This case–control-study included five extremely premature infants with small bowel VWM. In all 5 infants, birth weight was <600 g and gestational age at birth <26 weeks, and all were small for gestational age and female (for demographic variables see Table 1). In two infants, necrotic small bowel was resected after detorsion, leaving 55–65 cm of small intestine for recovery. In three infants, laparotomy revealed complete small bowel necrosis without apparent recovery following detorsion, and the abdomen was closed without resection. One of these infants died within hours of multi-organ failure, the other two had re-laparotomy confirming complete intestinal necrosis and died after a decision for palliative care.

**Table 1:** **Demographic variables**

	**Volvulus without malrotation**	**Matched controls**	**Necrotising enterocolitis**	**p-value**
**Number of infants (f/m)**	5 (5/0)	25 (12/13)	9 (7/2)	p = 0.506*^,#^
				p = 0.052**^,#^
**Gestational age at birth (weeks) (median (range))**	24.4 (23.6-25.4)	24.8 (23.1-26.4)	27 (23.6-33.6)	p = 0.402*
				p = 0.160**
**Birth weight (g) (median (range))**	480 (370–530)	570 (340–800)	950 (395–1460)	p = 0.048*^,##^
				p = 0.016**^,##^
**Weight at presentation (g) (median (range))**	970 (790–1180)		1145 (670–2470)	p = 0.64^##^
**Day of life at presentation (median (range))**	44 (37–52)		17 (2–97)	p = 0.028^##^
**Postmenstrual age at presentation (weeks) (median (range))**	31.0 (29.1-31.6)		33.1 (24.3-38.0)	p = 0.739^##^

f: female; m: male.

*: VWM-infants vs. NEC-infants.

**: VWM-infants vs. controls.

^#^: Fisher`s exact test, two tailed.

^##^: Wilcoxon test.

Demographic data of all 9 inborn infants who developed NEC within the same observational period are also depicted in Table 1.

**Figure 1:**
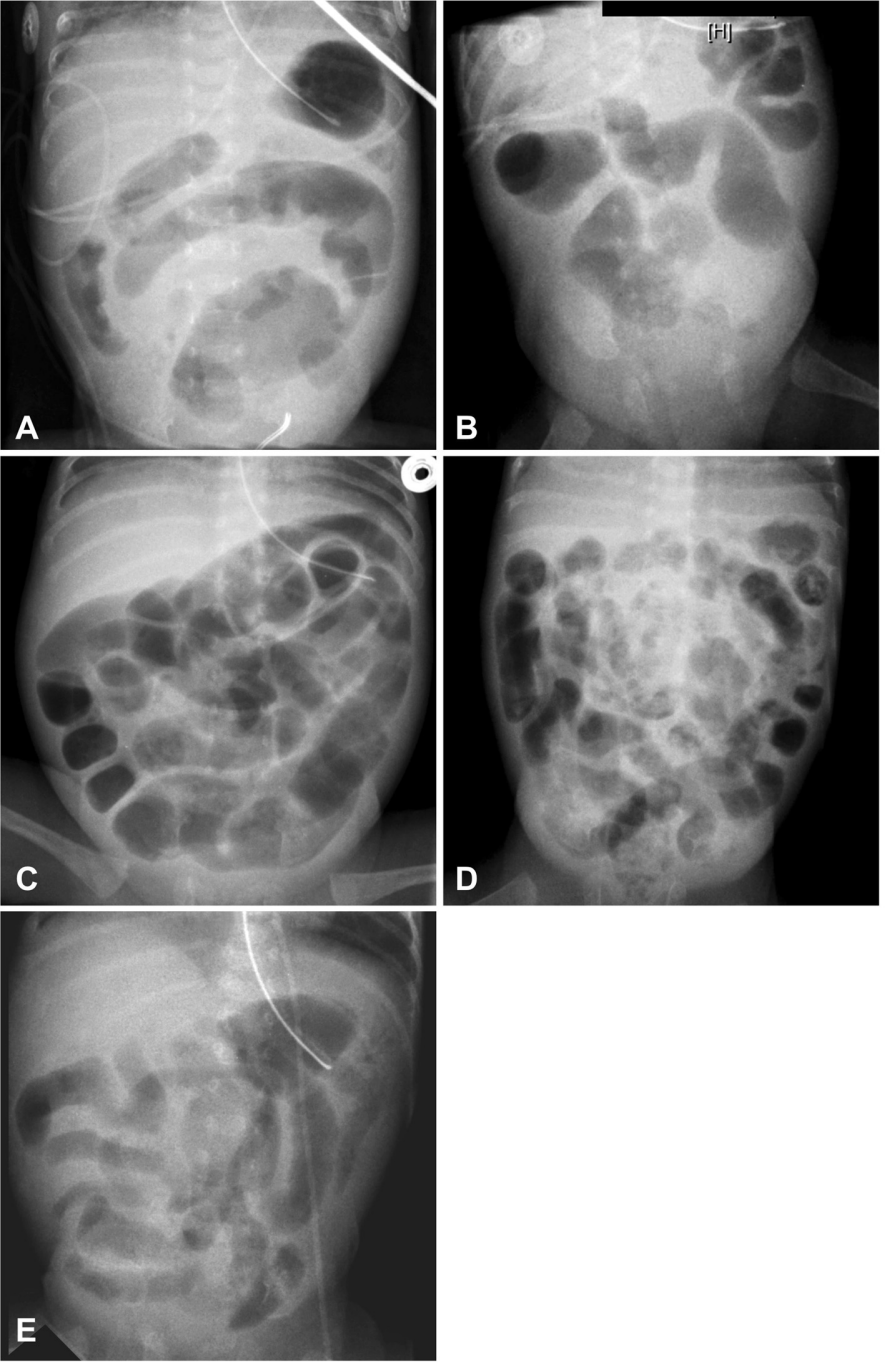
**Abdominal X-rays at clinical presentation of volvulus.** Abdominal X-rays at clinical presentation of volvulus: Panel **A** shows possibly suggestive concentric alignement of bowel loops, Panels **A**, **B**, **E** display unspecific intestinal wall thickening, Panel **D** may well have been compatible with necrotising enterocolitis and Panel **C** is unspecific.

Infants with VWM were significantly older at presentation than infants with NEC and their birth weight was lower (Table 1). However, neither postmenstrual age at birth nor postmenstrual age and weight at presentation differed significantly (Table 1). Furthermore, we found no striking differences in clinical signs or laboratory parameters between the two disease entities (Table 2). On the other hand, there was apparently a marked but possibly because of small numbers statistically non-significant difference in survival rate (2/5 VWM infants (40%) and 7/9 infants with NEC (78%), p = 0.27). Preoperative radiological findings in index infants were non-specific except for one patient who showed alignment of bowel loops compatible with volvulus (Figure 1A).

**Table 2:** **Clinical signs and selected laboratory parameters at day of presentation**

	**Volvulus without malrotation**	**Necrotising enterocolitis**	**p-value**
**Bile stained gastric residuals (n/N)**	4/5	6/9	p = 1.0*
**Blood in stool (n/N)**	1/5	3/9	p = 1.0*
**Emesis (n/N)**	1/5	3/9	p = 1.0*
Blood pH at presentation ***(median (range))	7.32 (6.6-7.37)	7.25 (7.00-7.34)	p = 0.64**
Base excess at presentation ***(median (range))	−6.6 (−32.0- -1.5)	−5.6 (−21.2-5.2)	p = 0.46**
Blood lactate at presentation ***(median (range))	3.3 (1.5-13.1)	2.5 (0.8-6.7)	p = 0.35**

*: Fisher`s exact test, two tailed.

**: Wilcoxon test.

***= all derived from venous blood samples.

Compared to matched control infants (n = 25), full enteral feeds were established earlier in VWM infants (Table 3). All VWM infants were almost exclusively fed their own mother’s milk supplemented with a standard multi-component fortifier. In the days before clinical deterioration, VWM-infants had significantly more frequent manipulations with rectal tubes (Table 3) for perceived meteorism, but there were no differences in the frequency of enemas, abdominal massage or defecation. Weight gain until VWM was not significantly different between index patients and their matched counterparts (SDS for weight at presentation – SDS for weight at birth = −0.15(−1.26/0.19) for VWM-infants and −0.43(−1.17/-0.17) for controls).

**Table 3:** **Variables of gastrointestinal function and manipulations intended to promote regular gastrointestinal transport**

	**Volvulus without malrotation**	**Controls**	**p-value by Wilcoxon-test**
**Last meconium evacuation (days) (median (range))**	5 (3–8)	7 (3–13)	p = 0.178
**Day of life, when full enteral feeds were reached (median (range))**	8 (6–10)	10 (6–30)	p = 0.039
**Number of enemas/d (d-3;d-1) (median** ^**#**^ **(range))**	0 (0–1)	0 (0–2)	p = 0.564
**Number of manipulations with rectal tubes/d (d-3;d-1) (median** ^**#**^ **(range; IQR))**	0 (0–3; 0–2)	0 (0–1; 0–0)	p = 0.047
**Frequency of abdominal massage/d (d-3;d-1) (median** ^**#**^ **(range))**	5 (4–6)	4 (0–6)	p = 0.240
**Number of stools/d (d-3;d-1) (median** ^**#**^ **(range))**	4 (3–6)	5 (3–7)	p = 0.647

(d-3;d-1): time period from three days until one day before presentation of volvulus or corresponding postnatal age in controls.

IQR: Interquartile range.

^#^= median of individual medians and range of individual medians are reported.

Full feeds were defined as ≥140 ml/kg/day of milk feeds actually administered for more than 72 h.

All infants (VWMs and controls) were on some nasal respiratory support (CPAP, nasal intermittent positive pressure ventilation (NIPPV) or nasal high-frequency-oscillation-CPAP (nHFO-CPAP)) at presentation of the abdominal event or at corresponding age, but significantly more infants in the VWM-group were on nasal high-frequency oscillation and there was a trend towards higher PEEP in the VWM-group (see Table 4).

**Table 4:** **Respiratory support**

	**Volvulus without malrotation**	**Controls**	**p-value**
**Duration of endotracheal mechanical ventilation until presentation/corresponding age (days) (median (range))**	7 (5–17)	7 (1–22)	p = 0.80**
**Nasal respiratory support (CPAP, NIPPV, nHFO-CPAP) at presentation/corresponding age (n/N)**	5/5	25/25	
**nHFO-CPAP at presentation/corresponding age (n/N)**	2/5	0/25	p = 0.023*
**Max. Peep at day of presentation/corresponding age (median (range; IQR))**	6.5 (6–8; 6.2-7.6)	6 (4–8; 5.9-6.3)	p = 0.078**

**: Wilcoxon test; *: Fisher`s exact test, two tailed; CPAP: continuous positive airway pressure; NIPPV: nasal intermittent positive pressure ventilation; nHFO-CPAP: nasal high-frequency-oscillation-CPAP.

IQR: Interquartile range.

## Discussion

To our knowledge, this is the first case–control-study investigating clinical signs and potential risk factors for VWM in extremely premature infants. We compared characteristics of VWM-infants with a cohort of matched extremely preterm infants and with all inborn VLBW infants who suffered from NEC during the same observational period. All infants had been treated according to the same institutional guidelines.

A striking finding and major trigger for initiating this study was the alarming mortality in our small cohort of infants with VWM. Whereas in other published case series in preterm infants the majority of infants survived [3,4,6], 3 out of 5 infants in our series eventually died. In comparison to other case series [3-6], infants in this study were considerable more immature (all <26 weeks at birth) and had substantially lower birth weights (range 370 – 530 g), these factors potentially leading to a significantly higher risk for a fatal course following VWM. Nonetheless, a conservative management in extremely premature infants with suspected NEC might be an additional factor contributing to an unedifying disease course as specific radiological or ultrasound features revealing the underlying strangulating obstruction leading to bowel ischemia in cases of VWM are missing [4].

In contrast to published case series, uniform growth restriction at birth and more pronounced immaturity in the present study are most striking. Consistent with previous case series, VWM predominantly occurred in girls and presented several weeks after birth. Particularly the “late-VWM-group” in the work of Drewett et al. presented at a very similar postnatal age (median age at presentation 45 days) [4].

Clinical presentation was that of an acute abdomen without any VWM-specific signs. Similarly, in earlier case series, there were also no specific clinical signs indicating VWM identified [3-5]. Just as previously reported [4,5], neither preoperative ultrasound nor radiological imaging was successful in detecting volvulus-specific features except for one patient in whom radiological findings were suggestive of volvulus (Figure 1A).

Unfortunately, there were no disease-specific clinical signs suitable for discriminating VWM and NEC. Thus, the dilemma between the necessity for prompt laparotomy to prevent irreversible intestinal ischemia in VWM and the preference for a conservative initial management of NEC persists in VLBW infants presenting with an acute abdomen. In the end, immediate surgical consultation in VLBW infants with sudden acute abdominal signs should be realized and prompt explorative laparotomy considered if the risk factors discussed below are present, the infant’s general condition deteriorates rapidly, and radiological and ultrasonographical signs that are more likely associated with NEC (such as portal venous gas, intramural gas) are absent. We appreciate that this recommendation contrasts with the widely practiced expectant management for suspected NEC but feel that there is no alternative for preventing bowel necrosis and death in case of (otherwise unrecognized) VWM.

## Potential risk factors for VWM

### Gender

In our case series only girls were affected by VWM. Billiemaz et al. reported 6 girls out of 7 affected infants [3], Zweifel et al. 2/3 [6]. Unfortunately, Drewett et al. [4] and Mark et al. [5] did not report the gender of affected children. Further population-based data are needed for a better assessment of this potential risk factor.

### Intestinal immaturity

Immaturity of intestinal motor function in very preterm infants is common, frequently leading to prolonged intestinal transit time and stasis of bowel contents [18,19]. Since stasis of bowel contents and long-standing subacute obstruction are discussed as key factors in the aetiology of VWM in preterm infants [4,12,20], one may speculate that extremely premature infants are at highest risk for developing VWM. This might apply even more for infants with a history of intrauterine growth restriction and potential intestinal hypoperfusion due to centralised prenatal circulation. All the more it is astonishing that all infants in our case series had an absolutely uneventful course of initial enteral feeding advancement, being completely enterally fed by day of life 8. In this series, VWM-infants reached full enteral feeds even earlier than matched controls. Additionally, no differences between VWM-infants and controls were observed regarding age at complete meconium evacuation and stool frequency prior to presentation.

### Manipulations for promoting regular gastrointestinal transport

The only difference between VWM-infants and controls in measures for promoting gastrointestinal transport (see Table 3) was the significantly more frequent use of rectal tubes, most likely for gaseous distension in the VWM-group in the days before presentation. It is difficult to imagine how this (insufficiently evaluated) practice could contribute to small intestinal volvulus. Hence, this observation might be a clue to more pronounced meteorism as an early but unspecific sign of emerging intestinal obstruction. Gentle abdominal massage is regularly performed in the vast majority of preterm infants in our unit, and we did not see a significant difference in the frequency of abdominal massage between VWM-infants and controls. Abdominal massage was accused of being a relevant risk factor for VWM in 2 uncontrolled case series [3,6], because many VWM cases received this intervention. Our case–control data, however, do not support this hypothesis. Although the practice of abdominal massage, rectal enemas or use of rectal tubes remained unchanged during and after the observational period, we did not observe any further infants with VWM for the following 2.5 years.

None of the infants investigated received erythromycin or any other prokinetic agent for promoting gastrointestinal transit prior to presentation.

### Respiratory support/Factors enhancing bowel distension

Respiratory support with CPAP is discussed as a risk factor for the development of VWM in preterm infants [4]. Nasal CPAP is well known to contribute to increased gaseous bowel distension in extremely premature infants (“CPAP belly syndrome”) [21,22]. Nevertheless, respiratory support via nasal/pharyngeal CPAP is widely applied in these infants at different stages of their respiratory illness because of its positive effects on short- and long-term respiratory outcomes.

It is conceivable that intestinal distension on top of immature intestinal motility might contribute to VWM. In our case series, all VWM-infants were on nasal CPAP or another form of nasal respiratory support at presentation. Yet, this was also true for all infants in the control group. Significantly more infants in the VWM-group had more intense respiratory support by nasal high-frequency oscillation-CPAP, although numbers are small; likewise PEEP levels tended to be higher in VWM infants. The increased use of rectal tubes in VWM patients may just reflect the increased abdominal distension associated with more intensive non-invasive respiratory support. Based on this observation, restriction of the use of nasal high-frequency oscillation may be considered and abdominal side effects of nasal ventilation modes other than CPAP should be carefully monitored and reported.

Formula feeding is commonly associated with delayed gastrointestinal transport and constipation in very preterm infants [23] and potentially could lead to increased bowel distension. However, all VWM-infants in our study almost exclusively received expressed (yet fortified) breast milk.

Appreciating the aforementioned potential risk factors linked to medical treatment in preterm infants after birth it is important to realize that VWM may occur in utero as well [7-9] without any preceding iatrogenic manipulation.

### Strengths and limitations of this study

In comparison to previously published case series, the case–control-design of our study enabled a more systematic evaluation of disease specific clinical signs of and risk factors for VWM. Nevertheless, this retrospective analysis based on a small number of index cases failed to identify strategies for the prevention of VWM.

## Conclusions

VWM in extremely premature infants represents a life-threatening event that typically occurs several weeks after birth with an acute abdomen. Female infants requiring intensive non-invasive (i.e., nasal) respiratory support with aggravated meteorism might be at highest risk, but a prospective population-based study is needed to learn more about the aetiology and risk factors for VWM in extremely premature infants.

## Abbreviations

VWM: Volvulus without malrotation
NEC: Necrotising enterocolitis
VLBW: Very low birth weight
SDS: Standard deviation score
NIPPV: Nasal intermittend positive pressure ventilation
nHF-CPAP: Nasal high-frequency-oscillation CPAP
